# Estimation of Uncertainty in Tracer Gas Measurement of Air Change Rates

**DOI:** 10.3390/ijerph7124238

**Published:** 2010-12-16

**Authors:** Atsushi Iizuka, Yumiko Okuizumi, Yukio Yanagisawa

**Affiliations:** 1 Research Center for Sustainable Science and Engineering, Institute of Multidisciplinary Research for Advanced Materials, Tohoku University, 2-1-1, Katahira, Sendai, Miyagi 980-8577, Japan; 2 Department of Environmental Systems, Institute of Frontier Sciences, The University of Tokyo, 5-1-5 Kashiwanoha, Kashiwa, Chiba 277-8563, Japan; E-Mails: yumiko_okuizumi@yy.k.u-tokyo.ac.jp (Y.O.); yukio@k.u-tokyo.ac.jp (Y.Y.)

**Keywords:** ventilation, tracer gas, indoor air, perfluorocarbon

## Abstract

Simple and economical measurement of air change rates can be achieved with a passive-type tracer gas doser and sampler. However, this is made more complex by the fact many buildings are not a single fully mixed zone. This means many measurements are required to obtain information on ventilation conditions. In this study, we evaluated the uncertainty of tracer gas measurement of air change rate in *n* completely mixed zones. A single measurement with one tracer gas could be used to simply estimate the air change rate when *n* = 2. Accurate air change rates could not be obtained for *n* ≥ 2 due to a lack of information. However, the proposed method can be used to estimate an air change rate with an accuracy of <33%. Using this method, overestimation of air change rate can be avoided. The proposed estimation method will be useful in practical ventilation measurements.

## 1. Introduction

Ventilation is an essential and effective countermeasure for reducing chemical pollution in indoor environments. Ventilation conditions are commonly evaluated by measuring the ventilation volume [m^3^/h], which is the uptake volume of outside air per unit time. An additional indicator is the air change rate [1/h], which is the ratio of the ventilation volume to volume of the indoor space. The air change rate is usually measured using a tracer gas. The main tracer gas methods use concentration decay, continuous emission, and steady concentration. In the concentration decay method, the tracer gas is released as a pulse into an indoor space and the decay of its concentration with time is continuously monitored. The air change rate can be estimated from the decay trend. This method is suitable for short term (up to several hours) monitoring of air change rate. In the continuous emission method, a constant amount of tracer gas is continuously emitted into an indoor space. The steady state concentration of the tracer gas is measured and used to calculate the air change rate. This method is generally used for long-term air change rate measurements. In the steady concentration method, the tracer gas concentration in the ventilated space is kept constant by controlling the emission rate of the gas. In this method, the variation in the ventilation rate with time is monitored. For all of these methods, complete mixing of the ventilated space is required to accurately estimate air change rate. For detailed discussions of these techniques, see Lagus and Persily [1], Serman [2], AIVC [3], or ASHRAE [4].

Passive-type tracer gas doser/sampler systems are a simple and cheap method for measurement of air change rate. Operation of the doser and sampler does not require any electric power, and it is simple enough for residents to operate themselves. Perfluorocarbon (PFC) is a common tracer gas used in these systems. Dietz and Cote [5] and Spengler *et al.* [6] developed ventilation measurements utilizing PFC. The technique has been researched and applied extensively in North America and Northern Europe [7,8]. In the United States, the Environmental Protection Agency (EPA) recommend methods utilizing PFT-CAT (Method IP4-A) or SF_6_ (Method IP4-B) for standard measurement of air change rates. However, measurements are more complex when many buildings do not consist of a single fully mixed zone. This means many measurements are required to obtain information on ventilation conditions.

The Conjunction Of Multizone Infiltration Specialists (COMIS) model [9] was developed to estimate ventilation conditions for multiple fully mixed zones. Input parameters for COMIS include air tightness and weather conditions. Yoshino *et al.* [10] reviewed ventilation measurements for multiple zones using various methods, such as the use of smoke to visualize airflow, and the application of four introduced tracer gases. Okuyama [11] used only one tracer gas to estimate air change rate for multiple zones. In this technique, the tracer gas emission rate was varied and changes in the tracer gas concentration were monitored. The data were used to statistically estimate the air change rate of the multiple zones. However, these methods are still complex, and for practical application simpler and more convenient ventilation measurements are required. Miller *et al.* [12] and Sherman [13] have discussed the estimation method for ventilation conditions for multi-zone building. Riffat *et al.* [14] reported the accuracy of single-tracer gas measurement for estimation of air flows between two zones.

In this study, we evaluated the measurement of air change rate for two fully mixed zones from a single measurement with one tracer gas. We discuss the uncertainty in this measurement for *n* fully mixed zones, and propose a simple method for estimation of air change rate for two fully mixed zones. This provides a simple method for practical application in ventilation studies.

## 2. Theory

### 2.1. Uncertainty in the Measurement of Air Change Rate with a Tracer Gas

**Figure 1:**
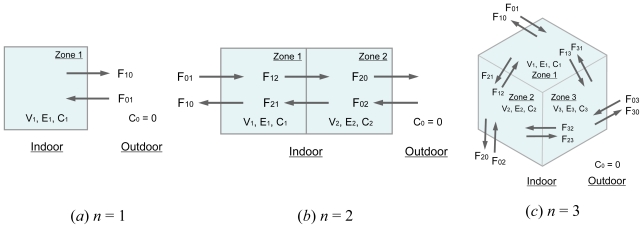
Schematic of buildings with 1, 2, or 3 fully mixed zones.

We first considered air change rate measurements in buildings of *n* fully mixed zones, where *n* is a natural number. The ventilation situations in buildings with 1–3 zones are illustrated in Figure 1. Measurements aim to determine the volume of air uptake from the outside into the *n* zones (air change rate). The uptake volume of air, *F_uptake_*, can be represented by the following equation:

(1) $$F_{uptake}=F_{01}+F_{02}+\cdots +F_{0n}=\sum_{i=1}^{n}F_{0i}$$

where *F*_0_*_i_* represents the air uptake volume to each zone. A single tracer gas is emitted at a known rate, *E_i_*, in zone *i*. The emitted tracer gas is collected by the passive-type air sampler and the average tracer gas concentration in zone *i*, *C_i_*, is measured. The volume of each zone, *V_i_*, is considered to be a known parameter as it can be measured. On the other hand, the volume of airflow from zone *i* to zone *j*, *F_ij_*, cannot be measured. All *F_ij_* have non-negative values. Generally, a gas that does not exist naturally outdoors is selected as the tracer gas, which means its concentration outside (zone 0), *C*_0_, is zero. Usually, the measurement time for the tracer gas method is sufficient that the tracer gas concentration is considered to be constant. In each zone, the air volume is also considered to be constant:

(2) $$\sum_{j=0}^{n}F_{ij}=\sum_{j=0}^{n}F_{ji}$$

This equation reflects the conservation of mass (air) in each zone.

If all fully mixed zones are in contact with each other and the air can freely pass between any two zones, then the number of unknown parameters *F_ij_* is *n*(*n* + 1). It should be noted that this provides the maximum number, and the actual number of unknown parameters is reduced compared with this when *F_ij_* = 0 before measurement. If zone *i* and *j* is not physically connected, *F_ij_* and *F_ji_* can be assumed as 0. A total of 2*n* balanced equations can be established, which includes tracer mass balance equations and air mass balance equations for the *n* fully mixed zones. Therefore, when calculating air change rate the information deficit is equal to *n*(*n* − 1) (= *n*(*n* + 1) − 2*n*). This is only equal to 0 when *n* = 1, and in all other cases we cannot obtain an accurate air change rate. Table 1 shows the maximum information deficit for *n* fully mixed zones.

To avoid this deficit, we have to supplement *n*(*n* − 1) data. For example, preparing and measuring the steady state concentrations of *n* types of tracer gases for *n* zones allows calculation of an accurate ventilation rate. Otherwise, with one tracer gas, *n* ventilation measurement trials are required to calculate the steady state concentration of the tracer gas in *n* zones. In this case, for each ventilation measurement, the location of the tracer gas doser is varied in *n* ways. However, these methods require either many tracer gases or many measurements, which means they cannot be practically applied. In addition, the time and money available for air change rate measurements are limited. Consequently, we evaluated the estimation of air change rate from a single measurement using one tracer gas.

### 2.2. Calculation When n = 1

As already mentioned, *F_uptake_* for a single fully mixed zone [Figure 1(a)] can be calculated accurately even from only a single measurement with one tracer gas. The tracer mass balance is given by:

(3) $$E_{1}+C_{0}F_{01}=C_{1}F_{10}$$

The indoor air mass balance is given by:

(4) $$F_{01}=F_{10}$$

where *E*_1_ is a known value and *C*_1_ is obtained by a tracer gas concentration measurement. *F_uptake_* can then be accurately calculated as follows:

(5) $$F_{fresh}=F_{01}=\frac{E_{1}}{C_{1}}$$

### 2.3. Calculation When n = 2

If building consists of two fully mixed zones [Figure 1(b)], it is more difficult to measure the air uptake *F_uptake_* than for *n* = 1. The tracer mass balance for the two fully mixed zones is as follows:

(6) $$V_{1}\frac{{dC}_{1}}{dt}=E_{1}+F_{01}C_{0}+F_{21}C_{2}-(F_{10}+F_{12})C_{1}$$

(7) $$V_{2}\frac{{dC}_{2}}{dt}=E_{2}+F_{02}C_{0}+F_{12}C_{1}-(F_{20}+F_{21})C_{2}$$

where *C_1_* and *C_2_* are considered to be constant after stabilization of tracer gas concentration. Therefore, we can transform the above equations to:

(8) $$E_{1}+C_{0}F_{01}+C_{2}F_{21}=C_{1}F_{10}+C_{1}F_{12}$$

(9) $$E_{2}+C_{0}F_{02}+C_{1}F_{12}=C_{2}F_{20}+C_{2}F_{21}$$

The air balance is represented by:

(10) $$F_{01}+F_{21}=F_{10}+F_{12}$$

(11) $$F_{02}+F_{12}=F_{20}+F_{21}$$

There are now six unknown parameters, *F*_01_, *F*_02_, *F*_10_, *F*_12_, *F*_20_, and *F*_21_ in the above four balanced equations, which means *F_uptake_*, which is defined by the following equation, cannot be calculated accurately:

(12) $$F_{uptake}=F_{01}+F_{02}$$

A situation such as this usually occurs with *n* ≥ 2, and the maximum information deficit is equal to *n*(*n* − 1). However, practically it is difficult to increase the number of tracer gases used or the number of measurements, which both consequently increase the measurement time. Therefore, it is necessary to estimate the ventilation volume for these zones.

## 3. Discussion

### 3.1. Estimation Method for Air Change Rate of Two Fully Mixed Zones from a Single Measurement with One Tracer Gas

For air change rate estimation of two fully mixed zones from limited information, we can utilize information about the volume of each zone, and tracer gas emission rates in each zone. Information about tracer gas concentrations in each zone can be obtained from passive air sampler measurements. As discussed in Section 2.3., four equations can be established for tracer gas mass balance and air balance for the two zones. There are six unknown parameters, *F*_01_, *F*_02_, *F*_10_, *F*_12_, *F*_20_, and *F*_21_, and *F_uptake_* cannot be accurately estimated. However, even in this case, we can estimate the air change rate with a practical level of accuracy using the following estimation method. This is due to the symmetry of Equations (8)–(12) and non-negative restrictions for the six unknown parameters.

Here, we use estimated air intake, *F_e_*, as an indicator of *F_uptake_*. *F_e_* is given by the sum of emission rates divided by the volume-weighted average tracer gas concentration, *C̄*:

(13) $$F_{e}=\frac{E_{1}+E_{2}}{\bar{C}}$$

where *C̄* is given by:

(14) $$\bar{C}=\frac{V_{1}}{V_{1}+V_{2}}C_{1}+\frac{V_{2}}{V_{1}+V_{2}}C_{2}$$

One of the purposes of the study is to show that *F_e_* is suitably accurate for practical applications and has a small range of error. The error between *F_e_* and *F_uptake_* can be indicated by the normalized parameter *y*:

(15) $$y=\frac{F_{e}-F_{uptake}}{F_{uptake}}$$

With non-negative restrictions on both parameters, the range of *y* is at least:

(16) y≥-1

Because *F_e_* and *F_uptake_* are not independent, the range of *y* is even more limited. A further parameter, *x*, can be introduced as an indicator of concentration differences between the two zones:

(17) $$x=\frac{C_{1}-C_{2}}{C_{1}+C_{2}}$$

With non-negative restrictions on *C_1_* and *C_2_*, the range for *x* is:

(18) -1≤x≤1

If the steady state concentration of tracer gas in each zone is equal, then the two zones can be treated as one fully mixed zone. So, when *x* = 0, *y* is 0, and the ventilation rate can then be accurately calculated. The range for y is mathematically more limited.

The restriction condition is non-negative restriction of six airflow volumes. There are six unknown parameters in the four balanced equations (Equations (8)–(11)), and we can express *y* as a function of two arbitrary unknown parameters. If *F*_10_ and *F*_20_ are selected as these two parameters, then *y* can be expressed as:

(19) $$y=\frac{\left(V_{1}+V_{2})\cdot (C_{1}F_{10}+C_{2}F_{20}\right)}{\left(C_{1}V_{1}+C_{2}V_{2})\cdot (F_{10}+F_{20}\right)}-1$$

If we select *F*_01_ and *F*_12_, then *y* can be expressed as:

(20) $$y=\frac{\left(E_{1}+E_{2})\cdot C_{2}\cdot (V_{1}+V_{2}\right)}{\left\{E_{2}+C_{2}F_{01}+(C_{1}-C_{2})F_{12}\}\cdot (C_{1}V_{1}+C_{2}V_{2}\right)}-1$$

and for *F*_02_ and *F*_21_:

(21) $$y=\frac{\left(E_{1}+E_{2})\cdot C_{1}\cdot (V_{1}+V_{2}\right)}{\left\{E_{1}+C_{1}F_{02}-(C_{1}-C_{2})F_{21}\}\cdot (C_{1}V_{1}+C_{2}V_{2}\right)}-1$$

It should be noted that only the symmetry of parameters was considered when selecting these three pairs, and other expressions would also give the same results.

In Equation (19), from non-negative restriction of *F*_10_ and *F*_20_, the range of *y* as a function of *x* can be calculated as follows:

(22) $$\text{For }x\geq 0,\;\;\;\frac{-2V_{1}\cdot x}{(V_{1}+V_{2})+(V_{1}-V_{2})\cdot x}\leq y\leq \frac{2V_{1}\cdot x}{(V_{1}+V_{2})+(V_{1}+V_{2})\cdot x}$$

(23) $$\text{For }x<0,\;\;\;\frac{2V_{1}\cdot x}{(V_{1}+V_{2})+(V_{1}-V_{2})\cdot x}\leq y\leq \frac{-2V_{1}\cdot x}{(V_{1}+V_{2})+(V_{1}+V_{2})\cdot x}$$

In Equation (20), from non-negative restrictions of *F*_01_ and *F*_12_, we can obtain the range of *y* as a function of *x* as follows:

(24) $$\text{For }x\geq 0,\;\;\;-1\leq y\leq \frac{-\{2E_{2}V_{1}+E_{1}(V_{1}+V_{2})\}\cdot x+E_{1}(V_{1}+V_{2})}{E_{2}(V_{1}-V_{2})\cdot x+E_{2}(V_{1}+V_{2})}$$

(25) For x<0,         -∞≤y≤∞

In Equation (21), from non-negative restrictions of *F*_02_ and *F*_21_, we can obtain the range of *y* as a function of *x* as follows:

(26) For x≥0,         -∞≤y≤∞

(27) $$\text{For }x<0,\;\;\;-1\leq y\leq \frac{\left\{2E_{1}V_{2}+E_{2}(V_{1}+V_{2})\}\cdot x+E_{2}(V_{1}+V_{2}\right)}{E_{1}(V_{1}-V_{2})\cdot x+E_{1}(V_{1}+V_{2})}$$

If non-negative restrictions for all six unknown parameters are considered, then combination of Equations (22)–(27) can accurately express the ranges for *y* (Appendices I–III). If *f*_1_(*x*) − *f*_4_ (*x*) are determined as follows:

(28) $$f_{1}(x)=\frac{-2V_{1}\cdot x}{(V_{1}+V_{2})+(V_{1}-V_{2})\cdot x}$$

(29) $$f_{2}(x)=\frac{2V_{1}\cdot x}{(V_{1}+V_{2})+(V_{1}+V_{2})\cdot x}$$

(30) $$f_{3}(x)=\frac{-\{2E_{2}V_{1}+E_{1}(V_{1}+V_{2})\}\cdot x+E_{1}(V_{1}+V_{2})}{E_{2}(V_{1}-V_{2})\cdot x+E_{2}(V_{1}+V_{2})}$$

(31) $$f_{4}(x)=\frac{\left\{2E_{1}V_{2}+E_{2}(V_{1}+V_{2})\}\cdot x+E_{2}(V_{1}+V_{2}\right)}{E_{1}(V_{1}-V_{2})\cdot x+E_{1}(V_{1}+V_{2})}$$

the ranges for *y* can be expressed as follows:

(32) $$\text{for }x\geq 0,\;\;\;f_{1}(x)\leq y\leq f_{2}(x)\;and\;y\leq f_{3}(x)$$

(33) $$\text{for }x<0,\;\;\;f_{2}(x)\leq y\leq f_{1}(x)\;and\;y\leq f_{4}(x)$$

### 3.2. Application of the Estimation Method

**Figure 2:**
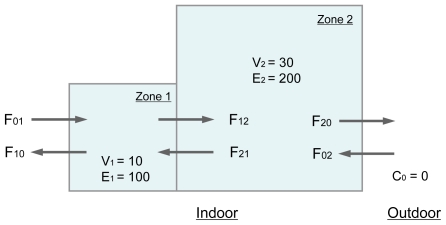
Schematic of a building consists of two fully mixed zones.

The estimation method can then be illustrated in application to a specific situation. It should be noted that the kind of tracer gas does not affect the results. This method is based on steady state. Thus, that requires constant airflow rates over a sufficient period of time for the concentrations to stabilize. Here, we attempt to simply measure the air change rate of two fully mixed zones. *V*_1_ and *V*_2_ are assumed to be 10 m^3^ and 30 m^3^, respectively. *E*_1_ and *E*_2_ are set as 100 μg/h and 200 μg/h, respectively. Figure 2 illustrates this situation. In the case, *f*_1_(*x*) − *f*_4_(*x*) were calculated as:

(34) $$f_{1}(x)=\frac{x}{x-2},f_{2}(x)=\frac{-3x}{x-2},f_{3}(x)=\frac{2x-1}{x-2},\;\text{and }f_{4}(x)=\frac{-7x-4}{x-2}$$

**Figure 3:**
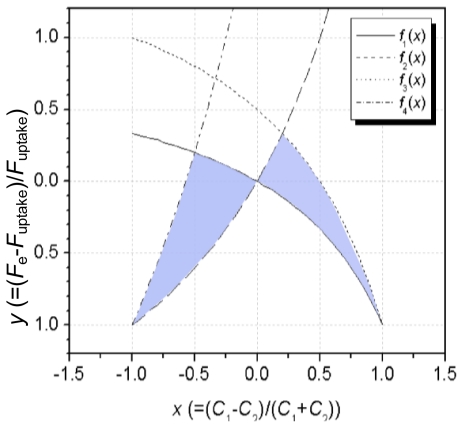
The range of *y* (colored area) when *V*_1_, *V*_2_, *E*_1_, and *E*_2_ are equal to 10 m^3^, 30 m^3^, 100 μg/h, and 200 μg/h, respectively.

Thus, the range of *y* is limited as shown in Figure 3. If *C*_1_ and *C*_2_ are measured to be 30 μg/m^3^ and 10 μg/m^3^, respectively, air intake *F_e_* can be estimated at 20 m^3^/h using Equations (13) and (14). In this case, *x* is calculated to be 0.5, so, *f*_1_ (0.5) = −0.33, *f*_2_ (0.5) = 1, and *f*_3_ (0.5) = 0. Thus, from Equation (32):

(35) -0.33≤y≤0

So, the estimated *F_e_* (20 m^3^/h) is accurate within 33%, and is an underestimate. From Equation 15:

(36) $$20\leq F_{uptake}\leq 30$$

**Figure 4:**
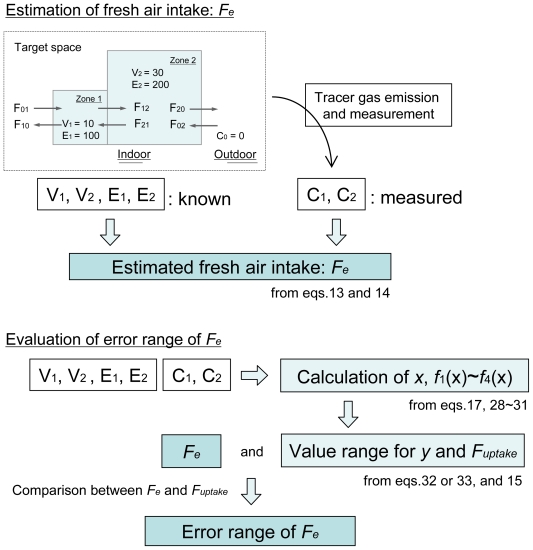
Flow chart for the estimation of air intake and its error range.

It should be noted that *F_e_* can be appropriately normalized in accordance with the aim of measurement. If we want to minimize the error rate, we can choose 25 ± 5 m^3^/h as *F*_e_ (error range is ±20%). To minimize the error rate, *E*_1_ and *E*_2_ should be set at the same value. In this case, the maximum error range of air intake *F*_e_ estimated by the methods is always <33%. This error value is equivalent to that reported by Riffat with single tracer gas measurement [14]. Miller *et al.* [12] reported that airflow rates in two-zone building could be estimated with an accuracy of 8% by two tracer gas decay experiments with the nonlinear least-squares minimization method in controlled conditions. One feature of this method is its simplicity in calculation operations. As described above, the proposed method can estimate air uptake volume with a practical accuracy only by using four arithmetic operations. If this estimation method can be combined with real time monitoring of a tracer gas concentration, we can extemporarily obtain the estimated air uptake without complex calculations. Furthermore, by using this method, overestimation of air change rate can be easily avoided. When aiming to avoiding overestimation of air change rate, the estimation method can give a minimum air change rate (in this case, *F_e_*_,min._ = 20 m^3^/h). Figure 4 summarizes the estimation flow chart for air intake and its error range.

## 4. Conclusions

In this study, we have evaluated the uncertainty in tracer gas measurement of air change rate for *n* fully mixed zones. We proposed a simple method to estimate the air change rate for two fully mixed zones from a single measurement using one tracer gas. Accurate air change rate could not be obtained for *n* ≥ 2 due to lack of information. However, the proposed method can provide an estimated air change rate with an accuracy of <33%. Using this method, overestimation of air change rate can be avoided. The proposed estimation method could find practical applications in ventilation studies.

## Figures and Tables

**Table 1:** The maximum information deficit for *n* fully-mixed zones.

Zone number	Maximum number of unknown parameters (I)	Obtainable information (II)	Maximum information deficit (III) = (I) – (II)
1	2	2	0
2	6	4	2
3	12	6	6
4	20	8	12
*n*	*n*(*n* + 1)	2*n*	*n*(*n* − 1)

## Appendices

### I. Derivation for Equations 22 and 23

From Equation (17):

(a-1) $$C_{2}=\frac{1-x}{1+x}\cdot C_{1}$$

Substitution of Equation (a-1) into Equation (19) yields:

(a-2) $$y=\frac{2\left\{\frac{-F_{20}}{F_{10}+F_{20}}\cdot V_{1}+\frac{F_{10}}{F_{10}+F_{20}}\cdot V_{2}\right\}\cdot x}{(V_{1}+V_{2})+(V_{1}-V_{2})\cdot x}$$

For a given *x*, from partial differentiation of Equation (a-2) by *F*_10_ and *F*_20_ we obtain:

(a-3) $$\frac{\partial y}{\partial F_{10}}=\frac{2x}{(V_{1}+V_{2})+(V_{1}-V_{2})\cdot x}\cdot \left\{\frac{(V_{1}+V_{2})\cdot F_{20}}{{\left(F_{10}+F_{20}\right)}^{2}}\right\}\geq 0$$

(a-4) $$\frac{\partial y}{\partial F_{20}}=\frac{2x}{(V_{1}+V_{2})+(V_{1}-V_{2})\cdot x}\cdot \left\{\frac{-(V_{1}+V_{2})\cdot F_{10}}{{\left(F_{10}+F_{20}\right)}^{2}}\right\}\leq 0$$

where *F*_10_ and *F*_20_ only have positive value, thus, *y*→*y*_max_ when *F*_10_→ +0, *F*_20_→ +∞, and *y*→ *y*_min_ when *F*_10_→ +∞, *F*_20_ → +0. Thus, we can obtain the range of *y* as a function of *x* as follows:

(22) $$\text{For }x\geq 0,\;\;\;\frac{-2V_{1}\cdot x}{(V_{1}+V_{2})+(V_{1}-V_{2})\cdot x}\leq y\leq \frac{2V_{1}\cdot x}{(V_{1}+V_{2})+(V_{1}+V_{2})\cdot x}$$

(23) $$\text{For }x<0,\;\;\;\frac{2V_{1}\cdot x}{(V_{1}+V_{2})+(V_{1}-V_{2})\cdot x}\leq y\leq \frac{-2V_{1}\cdot x}{(V_{1}+V_{2})+(V_{1}+V_{2})\cdot x}$$

### II. Derivation for Equations 24 and 25

*F*_01_ and *F*_12_ are only present in the denominator of Equation (20). When (*C*_1_ − *C*_2_) ≥0 (*x*≥ 0), minimization of the sum of *C*_2_*F*_01_ and (*C*_1_ − *C*_2_)*F*_12_ gives the maximum value for *y*. Thus:

(a-5) $$y_{\text{max}}=\frac{\left(E_{1}+E_{2})\cdot C_{2}\cdot (V_{1}+V_{2}\right)}{E_{2}\cdot (C_{1}V_{1}+C_{2}V_{2})}-1$$

From substitution of Equation (a-1) into Equation (a-5):

(a-6) $$y_{\text{mas}}=\frac{-\{2E_{2}V_{1}+E_{1}(V_{1}+V_{2})\}\cdot x+E_{1}(V_{1}+V_{2})}{E_{2}(V_{1}-V_{2})\cdot x+E_{2}(V_{1}+V_{2})}$$

The minimum value for *y* is obviously −1. Thus, for *x* ≥ 0:

(24) $$-1\leq y\leq \frac{-\{2E_{2}V_{1}+E_{1}(V_{1}+V_{2})\}\cdot x+E_{1}(V_{1}+V_{2})}{E_{2}(V_{1}-V_{2})\cdot x+E_{2}(V_{1}+V_{2})}$$

When (*C*_1_ − *C*_2_)<0(*x <* 0), the range of *y* only includes real numbers:

(25) For x<0,         -∞≤y≤∞.

### III. Derivation for Equations 26 and 27

*F*_10_ and *F*_21_ are only present in the denominator of Equation (21). When −(*C*_1_ − *C*_2_) <0 (*x* ≥ 0), the range of *y* only includes real numbers:

(26) For x≥0,         -∞≤y≤∞

When −(*C*_2_ − *C*_0_) ≥ 0(*x* < 0), minimization of the sum of *C*_1_*F*_02_ and −(*C*_1_ − *C*_2_)*F*_21_ gives the maximum value for *y*:

(a-5) $$y_{\text{max}}=\frac{\left(E_{1}+E_{2})\cdot C_{2}\cdot (V_{1}+V_{2}\right)}{E_{1}\cdot (C_{1}V_{1}+C_{2}V_{2})}-1$$

From substitution of Equation (a-1) into Equation (a-5),

(a-6) $$y_{max}=\frac{\left\{2E_{1}V_{2}+E_{2}(V_{1}+V_{2})\}\cdot x+E_{2}(V_{1}+V_{2}\right)}{E_{1}(V_{1}-V_{2})\cdot x+E_{1}(V_{1}+V_{2})}$$

The minimum value for *y* is obviously −1. Thus, for *x* < 0:

(27) $$-1\leq y\leq \frac{\left\{2E_{1}V_{2}+E_{2}(V_{1}+V_{2})\}\cdot x+E_{2}(V_{1}+V_{2}\right)}{E_{1}(V_{1}-V_{2})\cdot x+E_{1}(V_{1}+V_{2})}$$

## References

[1] Lagus P Persily AK A review of tracer-gas techniques for measuring airflows in buildings ASHRAE Transactions 1985 91 1075 1087

[2] Sherman MH Air infiltration measurement techniques Progress and Trends in Air Infiltration and Ventilation Research, Proceedings10th AIVC Conference, sponsored by the International Energy Agency Helsinki, Finland 25–28 September 1990 63 88

[3] AIVC Air Flow Patterns within Buildings. Measurement Techniques Technical Note 34 Air Infiltration and Ventilation Centre, Oscar Faber PLC St. Albans, UK 1991 Part III

[4] ASHRAE 1993 ASHRAE Handbook: Fundamentals American Society of Heating, Refrigerating and Air-Conditioning Engineers, Inc Atlanta, GA, USA 1993 Chapter 23

[5] Dietz RN Cote EA Air infiltration measurements in a home using a convenient perfluorocarbon tracer technique Environ. Int 1982 8 419 433

[6] Spengler JD Yanagisawa Y Ryan PB Manual for Infiltration Measurement Using Perfluorocarbon Traced Gas and Passive Collectors Harvard School of Public Health Boston, MA, USA 1985

[7] Pandian MD Ott WR Behar JV Residential air exchange rates for use in indoor air and exposure modeling studies J. Expo. Anal. Environ. Epidemiol 1993 3 407 416 8173341

[8] Pandian MD Behar JV Ott WR Wallace LA Wilson AL Colome SD Koontz M Correcting errors in the nationwide data base of residential air exchange rates J. Expo. Anal. Environ. Epidemiol 1998 8 577 586

[9] Fuestel HE COMIS—An international multizone air-flow and contaminant transport model, LBNL-42182 1998

[10] Yoshino H Matsumoto H Utsumi Y The measurement method of multizone infiltration (in Japanese) Journal of the Society of Heating, Air-Conditioning and Sanitary Engineering of Japan 1988 62 97 104

[11] Okuyama H State equation of general diffusion system using network concepts and theory of system parameter identification (in Japanese) Transactions of the architectural institute of Japan 1989 344 103 115

[12] Miller SL Leiserson K Nazaroff W Nonlinear least-squares minimization applied to tracer gas decay for determining airflow rates in a two-zone building Indoor Air 1997 7 64 75

[13] Sherman MH On the estimation of multizone ventilation rates from tracer gas measurements Build. Environ 1989 24 355 362

[14] Riffat SB Air flows between two zones: Accuracy of single-tracer gas measurements for estimation Building Serv. Eng. Res. Technol 1989 10 85 88

